# ^18^F-RGD PET/CT and Systemic Inflammatory Biomarkers Predict Outcomes of Patients With Advanced NSCLC Receiving Combined Antiangiogenic Treatment

**DOI:** 10.3389/fonc.2021.671912

**Published:** 2021-06-04

**Authors:** Jie Liu, Leilei Wu, Zhiguo Liu, Samuel Seery, Jianing Li, Zhenhua Gao, Jinming Yu, Xue Meng

**Affiliations:** ^1^Department of Radiation Oncology, Shandong Cancer Hospital and Institute, Shandong First Medical University and Shandong Academy of Medical Sciences, Jinan, China; ^2^Department of Medical Oncology, Cancer Center, Qilu Hospital of Shandong University, Jinan, China; ^3^Department of Humanities and Social Sciences, Peking Union Medical College, Chinese Academy of Medical Sciences and Peking Union Medical College, Beijing, China; ^4^Faculty of Health and Medicine, Division of Health Research, Lancaster University, Lancaster, United Kingdom

**Keywords:** ^18^F-RGD PET/CT, inflammatory biomarkers, outcome prediction, combined antiangiogenic therapy, NSCLC

## Abstract

**Background:**

The aim of this study was to evaluate ^18^F-AlF-NOTA-PRGD2 positron emission tomography/computed tomography (^18^F-RGD PET/CT) and serum inflammation biomarkers for predicting outcomes of patients receiving combined antiangiogenic treatment for advanced non-small cell lung cancer (NSCLC).

**Methods:**

Patients with advanced NSCLC underwent ^18^F-RGD PET/CT examination and provided blood samples before treatments commenced. PET/CT parameters included maximum standard uptake value (SUVmax) and mean standard uptake value (SUVmean), peak standard uptake value (SUVpeak) and metabolic tumor volume (MTV) for all contoured lesions. Biomarkers for inflammation included pretreatment neutrophil-to-lymphocyte ratio (PreNLR), pretreatment platelet-to-lymphocyte ratio (PrePLR), and pretreatment lymphocyte-to-monocyte ratio (PreLMR). Receiver operating characteristic (ROC) curve analysis was used to describe response prediction accuracy. Logistic regression and Cox’s regression analysis was implemented to identify independent factors for short-term responses and progression-free survival (PFS).

**Results:**

This study included 23 patients. According to ROC curve analysis, there were significant correlations between the SUVmax, SUVmean, and ^18^F-RGD PET/CT MTV and short-term responses (*p*<0.05). SUVmax was identified using logistic regression analysis as a significant predictor of treatment sensitivity (*p*=0.008). Cox’s multivariate regression analysis suggested that high SUVpeak (*p*=0.021) and high PreLMR (*p*=0.03) were independent PFS predictors. Combining SUVpeak and PreLMR may also increase the prognostic value for PFS, enabling us to identify a subgroup of patients with intermediate PFS.

**Conclusion:**

^18^F-RGD uptake on PET/CT and serum inflammation biomarker pretreatment may predict outcomes for combined antiangiogenic treatments for advanced NSCLC patients. Higher ^18^F-RGD uptake and higher PreLMR also appear to predict improved short-term responses and PFS. Combining biomarkers may therefore provide a basis for risk stratification, although further research is required.

## Introduction

Lung cancer remains the leading cause of cancer death, with 80-85% of the total number of lung cancer cases being non-small cell lung cancer (NSCLC) (1). Unfortunately, the vast majority of NSCLC patients are diagnosed late with local progression or evidence of metastasis (2). Bevacizumab combined with chemotherapy was initially approved by the United States Food and Drug Administration (US FDA) in 2006 as a first-line treatment for locally unresectable advanced, recurrent, metastatic, nonsquamous NSCLC due to evidence of a significant survival benefit and acceptable safety (3). However, not every patient benefits from this combination and so predictive markers are increasingly being used to identify NSCLC subpopulations who may benefit from receiving this combined intervention. Unfortunately, to date, research has been largely unsuccessful in identifying even a single response predictor for combined antiangiogenic treatments (4). Therefore, it remains necessary to identify predictive factors to ensure that this treatment is provided to those who will benefit most.

Integrin αvβ3, which forms complexes with vascular endothelial growth factor (VEGF) signaling pathways, is highly expressed on newly formed vessels (5, 6). The tripeptide sequence arginine-glycine-aspartate (RGD) also has a high affinity with and can therefore bind to integrin αvβ3. As such, ^18^F-AlF-NOTA-PRGD2 positron emission tomography/computed tomography (^18^F-RGD PET/CT) has been recommended for noninvasive angiogenesis imaging (7). ^18^F-RGD is a novel tracer that one-step labels integrin αvβ3, which targets the PET probe, and has proven relatively safe (8). In a pilot clinical study, patients with lesions broader than ^18^F-RGD PET/CT parameters appeared to respond better to antiangiogenic drugs alone (9). These initial studies validated some of the underlying theories that are emerging within this field and further suggest the need to explore the predictive capacity of ^18^F-RGD PET/CT for combining antiangiogenic and chemotherapeutic interventions.

Serum inflammatory cells have a complex relationship with angiogenesis and can be used to reflect the immune system status by providing insight into the tumor microenvironment (10). In particular, neutrophil and monocyte subpopulations have been found to contribute to angiogenesis (11, 12). However, activated T cells appear to inhibit neoangiogenesis by releasing the immune-related cytokine IFN-γ (4). Antiangiogenic therapies with bevacizumab may therefore increase the infiltration of immune effector cells and convert an intrinsically immunosuppressive tumor microenvironment into an immunosupportive microenvironment (13). Here, we propose that systemic immune factors, including the pretreatment neutrophil-to-lymphocyte ratio (PreNLR), pretreatment platelet-to-lymphocyte ratio (PrePLR), and pretreatment lymphocyte-to-monocyte ratio (PreLMR), may be useful predictors for combined antiangiogenic treatments. As such, we investigated whether these ^18^F-RGD PET/CT parameters with systemic immune factors could be used as predictive markers for combined antiangiogenic treatments.

## Materials and Methods

### Patients and Eligibility

**Figure 1:**
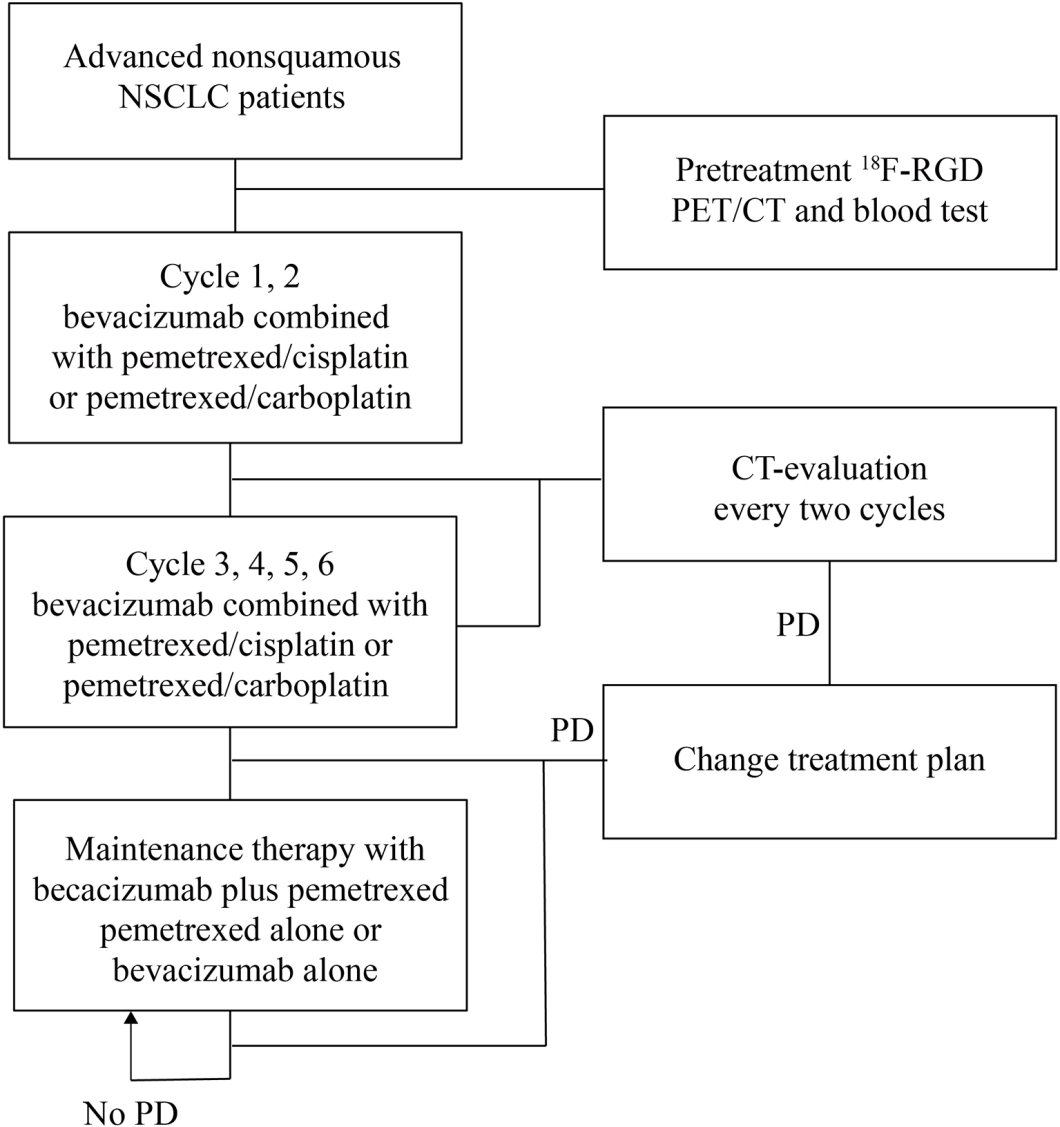
Study design.

In this prospective study, 30 patients with advanced nonsquamous NSCLC were initially enrolled. Each patient had been diagnosed through histologic examination and had pretreatment ^18^F-RGD PET/CT scans. This study involved patients from Shandong Cancer Hospital over a one-year period, from December 2018 to December 2019. To be included, patients had to be at least 18 years of age with a Karnofsky performance status (KPS) of ≥70. Patients who did not have objectively measurable lesions or those with autoimmune diseases or active comorbid infections were excluded. A total of 23 patients were included in the final analysis. Formal consent was requested and received from all prospective candidates prior to participation. This study was approved by the Institutional Review Board and the ethics committee within Shandong Cancer Hospital (reference no. SDTHEC 20180311). A flow chart of the study design is provided as **Figure 1**.

### Treatment Regimen

Patients received bevacizumab combined with pemetrexed/cisplatin or pemetrexed/carboplatin every three weeks. Those who did not progress during four or six therapeutic cycles were assigned to continuous maintenance therapy with bevacizumab plus pemetrexed, pemetrexed alone, or bevacizumab alone, according to treatment plans devised by a patient’s physician. Treatments continued until there was evidence of progressive disease (PD) according to RECIST criteria (v.1.1) or intolerable toxicity or patients requested to withdraw from further participation. Only one patient with advanced squamous cell carcinoma was treated with endostar plus docetaxel/carboplatin. This patient returned to hospital regularly for follow-ups after six treatment cycles.

### ^18^F-RGD PET/CT Scanning

Baseline ^18^F-RGD PET/CTs were performed within five days prior to the commencement of treatment. Lyophilized kits for labeling PRGD2 peptides were purchased from Jiangsu Institute of Nuclear Medicine, and synthesis progress was performed according to the standards described in a previous related study (14).

Radiochemical ^18^F-RGD purity exceeded 95%, and specific radioactivity exceeded 37 GBq (1,000 mCi)/μmol. Patients were required to rest for approximately one hour after receiving intravenous ^18^F-RGD injections at 219.24 ± 25.7 MBq. Patients were then asked to remain calm and sustain slow breathing during image acquisition. PET images were acquired from head to thigh (with patients in a supine position) for five minutes for each perspective, with an axial sampling thickness of 4.25 mm per slice after intravenous administration of ^18^F-RGD.

Images were attenuated with transmission data from the CT scans. This device was utilized because it is capable of multi-slice helical CT for anatomic imaging and attenuation correction. Attenuation-corrected PET images, CT images, and fused PET/CT images are presented as coronal, sagittal and transaxial slices and were viewed through a Xeleris workstation (GE Healthcare).

### Image Analysis

Two qualified, experienced physicians analyzed the PET/CT images using MIM software (MIM, 6.1.0, Ohio, USA) without knowledge of the patients’ respective histories. Significant differences between the physicians opinions in terms of regions of interest (ROIs) and tumor uptake values, were discussed until consensuses were reached. ROIs were drawn around lesions, referencing anatomical structures according to CT images and PET/CT fused images for ROI accuracy. ROIs were defined as a closed area in the image, which usually has similar characteristics. In this study, ROIs were the anatomical structures of tumors from CT images, which help to acquire the tumor uptake value of ^18^F-RGD.

The maximum standard uptake value (SUVmax) and mean standard uptake value (SUVmean) of all tumors were generated using a vendor-provided automated contouring program based on a 2.5 threshold. The peak standard uptake value (SUVpeak) was acquired as the average SUV within a one cubic centimeter sphere surrounding the voxel with the SUVmax. Metabolic tumor volume (MTV) was measured through attenuation-corrected ^18^F-RGD PET images using an SUV-based automated contouring program with an iso-counter threshold method based on 41% of the SUVmax, defined as the total volume of all tumors in the body in milliliters.

### Inflammatory Factors

Complete blood counts were obtained three days prior to the start of treatment and were all performed in the Department of Clinical Laboratory within Shandong Cancer Hospital. Inflammatory factors, including baseline NLR, PLR and LMR. These baseline data were abstracted from patients’ records and then anonymized. The NLR was calculated by dividing the absolute neutrophil count by the absolute lymphocyte count. The PLR was calculated by dividing the absolute platelet count by the absolute lymphocyte count. Likewise, the LMR was calculated by dividing the absolute lymphocyte count by the absolute monocyte count.

### Study Endpoint

Short-term treatment responses were assessed after the 2^nd^ cycle using the treatment response evaluation according to RECIST criteria (v.1.1) using CT. Patients with a complete response (CR) or partial response (PR) were classified as ‘responders’. Those who appeared with stable disease (SD) or PD were defined as ‘nonresponders’. Progression-free survival (PFS) was measured from the start of treatment until the date when disease progression was ascertained or in the occurrence of death due to any cause. Patients who died without documented PD were considered to have had PD at the time of death.

### Statistical Analyses

All statistical tests were performed with SPSS 23.0 (SPSS Inc., Chicago, IL) and R software (version 3.6.2). Quantitative data are expressed as the means with corresponding standard deviations (SD). Two-sample *t*-tests and Wilcoxon rank-sum tests were used to compare PET/CT parameters and inflammatory factors between responders and nonresponders. ROC curves and area under the ROC curve (AUC) analyses were used to describe response prediction accuracy. Logistic regression analysis was applied to identify correlations between variables and short-term outcomes. Cox’s proportional hazard regression model was utilized to investigate the independence of survival and other related factors. Variables with *p*<0.1 under univariate analysis were entered into Cox’s multivariate regression to build prognostic models.

The results are presented as hazard ratios (HRs) with corresponding 95% confidence intervals (95% CIs). Continuous prognostic variables were dichotomized for PFS before Kaplan-Meier analysis using optimal cutoff values determined using the “surv_cutpoint” function in the “survminer” package in R. Correlations between PET parameters and inflammatory factors were calculated using Spearman’s rank correlation. All variables with *p*<0.05 were considered statistically significant.

## Results

### Patients and Short-Term Outcomes

**Figure 2:**
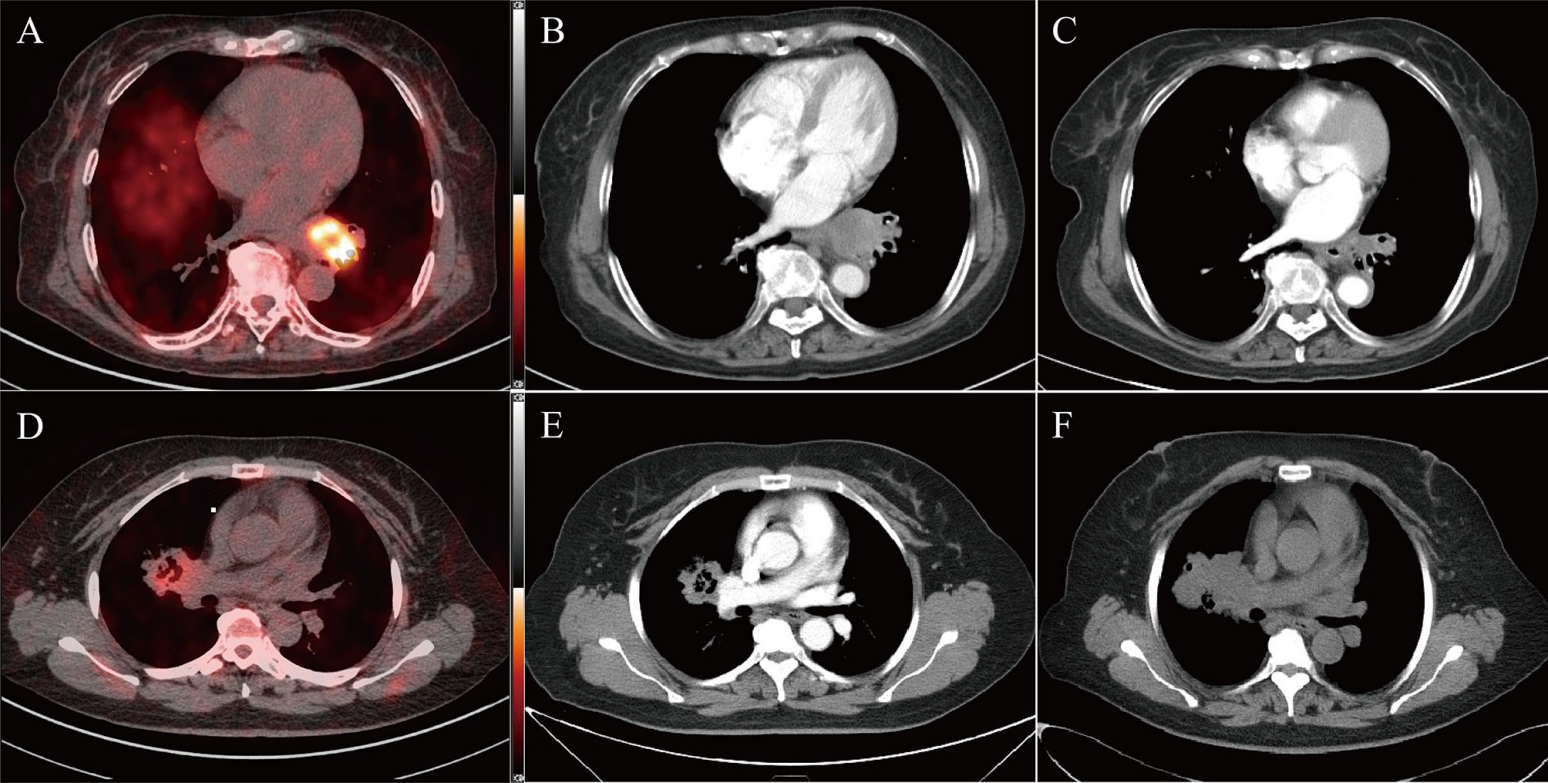
Two typical examples of ^18^F-RGD PET/CT scans in responder (top panel, SUVmax = 7.99, SUVmean = 3.39, MTV = 5.02, Response evaluated as PR) and non-responder (bottom panel, SUVmax = 3.38, SUVmean = 2.76, MTV = 2.07, Response evaluated as PD). Baseline PET/CT images **(A, D)** and corresponding CT slices from before treatment **(B, E)** and 2 cycles after treatment **(C, F)**.

23 patients were included for complete short-term response and survival analyses. Seven patients were excluded from the original 30 eligible participants, four of whom refused combined antiangiogenic therapy with chemotherapy, and three discontinued treatment after the first cycle. One patient died from bleeding caused by tumor-infiltrating blood vessels before response assessment. Therefore, 22 patients were included in this short-term response analysis. Among these patients, nine were assessed as PR, and the overall response rate was 40.91%. By contrast, 11 patients achieved SD, and two patients attained PD. Patient characteristics are summarized in **Table 1** and listed in **Table 2** and **Supplementary Table 1**. ^18^F-RGD PET/CT and CT scans for a typical responder and nonresponder are presented in **Figure 2**.

**Table 1:** Patient characteristics.

Characteristics		Participants (N=23)	Percentage (%)
Age			
	<60	7	30.43
	≥60	16	69.57
Gender			
	Male	15	65.22
	Female	8	34.78
Tumor stage			
	IIIB/C	4	17.39
	IVA/B	19	82.61
Histopathologic subtype			
	Adenocarcinoma	22	95.65
	Squamous cell carcinoma	1	4.35
Smoking history			
	Yes	12	52.17
	No	11	47.83
KPS			
	≤80	14	60.87
	>80	9	39.13
Number of treatment regimens			
	1	17	73.91
	2	6	26.09

*N denotes whole sample.

**Table 2:** Patients characteristics, outcomes, tumor pre-treatment ^18^F-RGD PET uptake and inflammatory biomarkers.

Patient no.	Gender	Age(y)	Tumor stage	Smoking history	No. of treatment regimens	Histology	EGFR	Pretreatment ^18^F-RGD PET uptake				PreNLR	PrePLR	PreLMR	2 Cycles Response	Time to progression (mo)
								SUVmax	SUVmean	SUVpeak	MVT					
1	M	59	IIIB	Former	1	Adenocarcinoma	Negative	2.61	1.32	2.05	45.95	1.73	162.57	3.73	NA	0.37
2	F	42	IIIC	None	1	Adenocarcinoma	Not available	5.29	3.34	4.24	17.50	2.44	204.98	5.58	SD	8.43
3	M	68	IVB	Former	1	Adenocarcinoma	Negative	2.80	2.58	2.36	0.44	20.53	776.36	0.38	PD	1.77
4	F	73	IVB	None	1	Adenocarcinoma	Not available	7.52	3.42	5.58	12.67	1.92	167.62	2.14	SD	6.87
5	M	68	IVB	Former	2	Adenocarcinoma	Negative	3.66	2.70	3.23	19.31	2.68	127.63	2.62	SD	3.23
6	M	69	IIIC	Former	1	Squamous cell carcinoma	Not available	7.01	3.57	5.61	43.97	2.34	129.07	3.74	SD	14.57+
7	M	69	IVB	None	2	Adenocarcinoma	Not available	12.92	8.59	3.39	394.97	2.40	120.10	6.69	PR	10.00
8	M	35	IVA	None	1	Adenocarcinoma	Sensitive mutations	4.59	3.02	3.84	21.45	2.78	214.11	4.29	PR	14.13+
9	F	52	IVB	None	1	Adenocarcinoma	Negative	2.86	2.60	2.36	7.52	3.02	185.08	1.35	SD	5.93
10	M	52	IVB	Former	1	Adenocarcinoma	Negative	3.90	2.93	3.00	12.30	5.47	337.50	1.75	SD	2.80
11	F	66	IVB	None	1	Adenocarcinoma	Negative	4.23	2.86	3.65	23.25	2.88	163.53	2.66	PR	5.27
12	M	61	IVA	Former	2	Adenocarcinoma	Sensitive mutations	4.21	2.98	3.64	30.29	5.04	157.55	1.28	PR	6.20
13	M	64	IVB	Former	1	Adenocarcinoma	Negative	5.34	3.06	3.99	25.37	3.86	255.08	1.76	PR	5.83
14	F	56	IVA	None	2	Adenocarcinoma	Sensitive mutations	4.21	2.91	3.17	2.82	2.69	219.66	4.50	SD	7.70
15	F	62	IVB	None	1	Adenocarcinoma	Not available	4.18	3.03	3.71	22.63	3.66	269.23	2.10	SD	7.80
16	M	62	IIIC	Former	2	Adenocarcinoma	Sensitive mutations	3.84	2.86	3.00	8.20	7.00	433.33	1.46	SD	3.60
17	M	60	IVB	Former	2	Adenocarcinoma	Negative	3.93	2.86	2.98	2.32	1.73	66.90	3.09	SD	3.50
18	M	69	IVB	Former	1	Adenocarcinoma	Negative	6.28	3.10	5.38	75.49	3.22	146.91	2.98	PR	5.53
19	F	66	IVB	None	1	Adenocarcinoma	Negative	3.38	2.76	2.71	2.07	4.17	396.15	2.23	PD	2.07
20	M	68	IVB	Former	1	Adenocarcinoma	Negative	4.59	3.00	3.55	2.40	5.31	125.96	2.30	SD	4.23
21	M	31	IVB	None	1	Adenocarcinoma	Negative	6.83	3.08	5.40	23.29	12.47	424.29	5.00	PR	6.27+
22	M	66	IVB	Former	1	Adenocarcinoma	Negative	11.03	3.98	8.83	166.50	7.38	262.50	0.67	PR	6.10+
23	F	65	IVB	None	1	Adenocarcinoma	Negative	7.99	3.39	5.02	45.75	2.89	284.58	2.46	PR	2.87

PreNLR, pre-treatment neutrophil-to-lymphocyte ratio; PrePLR, pre-treatment platelet-to-lymphocyte ratio; PreLMR, pre-treatment lymphocyte-to-monocyte ratio; PD, progressive disease; PR, partial response; SD, stable disease; NA, not applicable.

### Survival Outcome

Median follow-up time was 12 months (range 7.08-16.92). The median PFS (mPFS) was 5.83 months (range 3.17-8.49). The 6-month actuarial PFS rate was 43.48%. During follow-up, four patients were still alive without known recurrent disease. Seven patients had locoregional recurrence or metastasis but were still alive after salvage or palliative treatment. Four patients died of tumor recurrence, six patients died of distant organ metastasis, including metastasis to the brain and lung, and one patient died of both tumor recurrence and distant organ metastasis. Another patient died of bleeding caused by tumor-infiltrating blood vessels.

### Predictors of Short-Term Responses Among ^18^F-RGD PET/CT, Inflammatory Biomarkers, and Clinical Parameters

**Figure 3:**
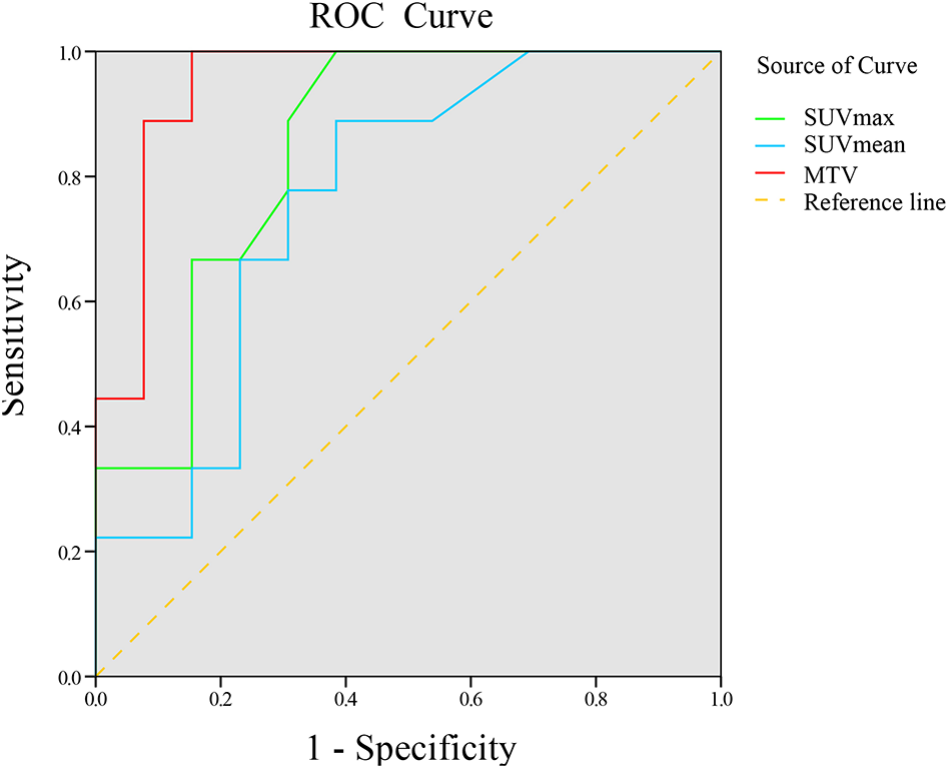
ROC curve of ^18^F-RGD PET/CT parameters to predict tumor response to combined anti-angiogenesis with chemotherapy.

^18^F-RGD PET/CT and inflammation parameters are provided in **Table 3**. The SUVmax, SUVmean, and MTV were significantly higher in responders compared to nonresponders (p<0.05). ROC curve analysis suggested that there were significant correlations among SUVmax, SUVmean, and MTV and 18F-RGD PET/CT and short-term responses (p<0.05). Additional details can be found in **Figure 3**.

**Table 3:** Pretreatment ^18^F-RGD PET/CT and inflammatory biomarkers for patients, and AUC curve analysis for predicting tumor responses.

Parameters	All patients	Responders	Non-responsers	P	ROC Curve Analysis					
					Area	P	Threshold	Sensitivity	Specificity	Accuracy
SUVmax	4.41 ± 2.57	6.28 ± 3.10	3.93 ± 1.44	0.030	0.846 ± 0.083	0.007	4.195	100	61.5	77.3
SUVmean	3.01 ± 1.23	3.08 ± 1.83	2.91 ± 0.31	0.036	0.761 ± 0.103	0.042	2.955	88.9	61.5	72.7
SUVpeak	3.65 ± 1.47	3.99 ± 1.70	3.17 ± 1.06	0.287	–	–	–	–	–	–
MTV	20.38 ± 86.23	30.29 ± 123.68	8.20 ± 12.10	0.004	0.949 ± 0.047	0.000	20.38	100	84.6	90.9
PreNLR	3.12 ± 4.27	3.22 ± 3.28	3.02 ± 4.97	0.969	–	–	–	–	–	–
PrePLR	209.54 ± 155.69	214.11 ± 94.29	204.98 ± 189.24	0.547	–	–	–	–	–	–
PreLMR	2.38 ± 1.61	2.66 ± 1.92	2.23 ± 1.39	0.461	–	–	–	–	–	–

PreNLR, pre-treatment neutrophil-to-lymphocyte ratio; PrePLR, pre-treatment platelet-to-lymphocyte ratio; PreLMR, pre-treatment lymphocyte-to-monocyte ratio.

^18^F-RGD PET/CT parameters, including the SUVmax, SUVmean, and MTV, and multiple clinical variables combined with the biomarkers of inflammation were tested through binary logistic regression analysis. According to univariate analysis, SUVmax and MTV could predict short-term outcomes (*p*<0.05), although SUVmean could not (*p*=0.396). Multivariate analysis revealed that SUVmax was a predictor of sensitivity to the combined antiangiogenic treatment (*p*=0.008).

### Independent Predictors of PFS Among ^18^F-RGD PET/CT, Inflammatory Biomarkers, and Clinical Parameters

^18^F-RGD PET/CT parameters, including SUVmax, SUVmean, SUVpeak and MTV, as well as multiple clinical variables and biomarkers for inflammation in relation to PFS, were further investigated using univariate Cox regression analysis. As shown in **Table 4**, SUVpeak (*p*=0.015) and PreLMR (*p*=0.030) were significantly associated with PFS.

**Table 4:** Univariate and multivariate progression-free survival analyses for patients with advanced NSCLC.

Variables	PFS						
	Univariate analysis				Multivariate analysis		
	HR	95%CI	P value		HR	95%CI	P value
Age	1.031	0.988-1.077	0.157				
Gender	1.227	0.480-3.137	0.670				
Smoking History	0.493	0.193-1.258	0.139				
Anatomical location	1.883	0.620-5.713	0.264				
EGFR mutation	0.881	0.291-2.670	0.823				
KPS	0.933	0.850-1.024	0.144				
No. of treatment lines	1.531	0.599-3.908	0.373				
Treatments procedures	1.593	0.367-6.921	0.535				
Tumor size	1.048	0.923-1.191	0.462				
Tumor stage	1.447	0.166-12.658	0.736				
SUVmax	0.766	0.583-1.006	0.055				
SUVmean	0.651	0.332-1.274	0.210				
SUVpeak	0.438	0.268-0.870	0.015		0.535	0.315-0.910	0.021
MTV	0.997	0.991-1.003	0.292				
Pre-NLR	1.072	0.933-1.232	0.327				
Pre-PLR	1.003	0.999-1.007	0.089				
Pre-LMR	0.684	0.486-0.965	0.030		0.675	0.474-0.963	0.030

No., number; PreNLR, pre-treatment neutrophil-to-lymphocyte ratio; PrePLR, pre-treatment platelet-to-lymphocyte ratio; PreLMR, pre-treatment lymphocyte-to-monocyte ratio.

**Figure 4:**
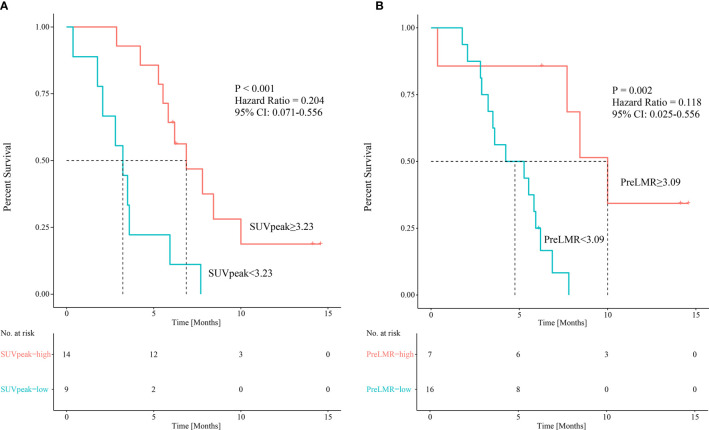
Kaplan-Meier curves showing patients’ PFS among patients with different SUVpeak **(A)** and PreLMR levels **(B)**.

Variables considered highly significant with a *p*<0.1 were then intercalated into a multivariate regression model. SUVpeak (*p*=0.021) and PreLMR (*p*=0.030) were verified as independent prognostic factors for PFS. Patients with SUVpeak >3.23 and PreLMR >3.09 experienced prolonged PFS (HR=0.204, 95% CI: 0.071-0.556, p<0.001; HR=0.118, 95% CI: 0.025-0.556, p=0.002). Further details are provided in **Figure 4**.

### Combining the SUVpeak and PreLMR Parameters

**Figure 5:**
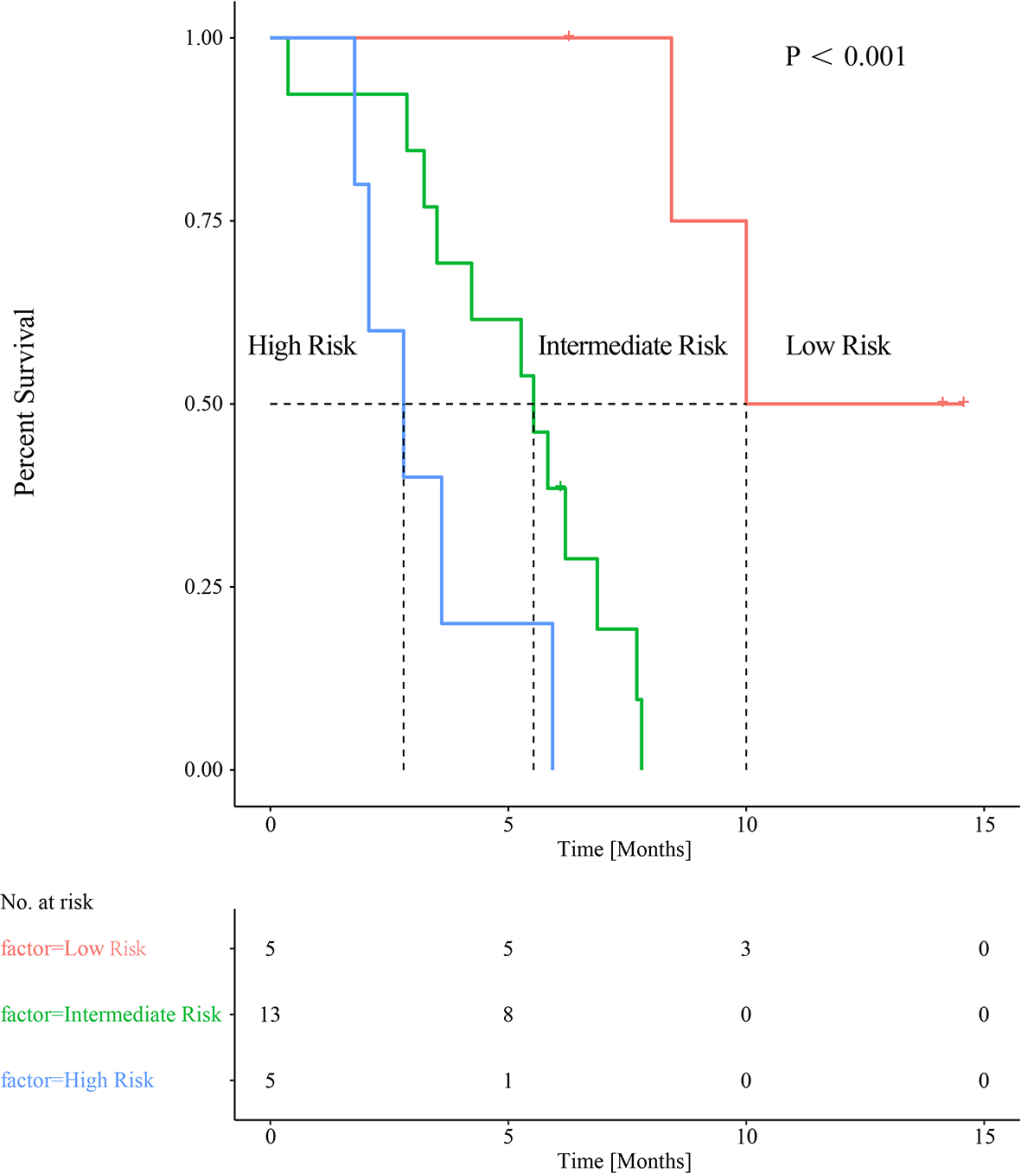
Combined baseline SUVpeak and PreLMR resulting in categorization into three distinct groups of patients with significantly different median PFS.

Baseline SUVpeak appears not to correlate significantly with PreLMR (Spearman rank correlation 0.057, *p*=0.341). When applying the respective cutoff values, we combined SUVpeak with PreLMR to stratify patients into three risk groups: high-SUVpeak patients with a high PreLMR (low-risk group; mPFS=10 months, n=5); high-SUVpeak patients with a low PreLMR and low-SUVpeak patients with a high PreLMR (intermediate-risk group; mPFS=5.53 months, n=13; low-risk group vs. intermediate-risk group *p*=0.001); and low-SUVpeak patients with a low PreLMR (high-risk group; mPFS=2.80 months, n=5; low-risk group vs. high-risk group *p*=0.02; intermediate-risk group vs. high-risk group, *p*=0.049). See **Figure 5** for further details.

## Discussion

This prospective study provided the first evidence that tumor ^18^F-RGD uptake parameters and systemic biomarkers for inflammation at baseline can be used to predict outcomes in patients receiving combined antiangiogenic therapy for advanced NSCLC. The tumor SUVmax obtained from ^18^F-RGD PET/CT was significantly higher in responders than nonresponders. Patients with tumors with higher SUVpeak or higher PreLMR had comparatively longer PFS. Although without a linear correlation, the combination of SUVpeak and PreLMR may increase the prognostic value and enable the identification of a subgroup of patients with an intermediate PFS. The exclusion of the seven patients did not change the prognostic value of these parameters since the included patients received a standard treatment.

A large proportion of the included patients had received two prior combined antiangiogenic treatments (26.09%). This may be the reason why we observed a median PFS slightly lower than 6.2 months in the bevacizumab combined treatment arm in the ECOG4599 trial. For tumors with high microvessel density (MVD), anti-angiogenic drugs are more likely to induce blood vessel normalization, thereby increasing the effect of anti-tumor treatment, which in turn makes the tumor insensitive to treatment. Therefore, the baseline MVD level has the potential to predict the efficacy of anti-angiogenic therapy. However, immunohistochemistry of tumor is an invasive test, and the heterogeneity of the tumor is likely to cause unstable results. Therefore, molecular imaging and hematology markers are expected to become potential indicators.

In a previous preclinical study, we found that the degree of tumor responses to bevacizumab alone, apatinib alone, or bevacizumab combined with chemotherapy or radiotherapy was positively associated with ^18^F−RGD tumor uptake prior to treatment (15). ^18^F-RGD uptake decreased more in high-uptake tumors than in low-uptake tumors. Several other preclinical studies have found that RGD uptake from pre- to post-antiangiogenic monotherapy decreased more than that in the control groups, and this occurs much earlier than the fluorodeoxyglucose (FDG) metabolic response (16). From a preclinical perspective, tumor RGD uptake at baseline or RGD uptake changes in the early stage seems to be a strong predictor for antiangiogenic therapies.

In a previous clinical study with bevacizumab-containing therapy in ovarian and cervical cancers, larger decreases in the SUVmean related to RGD uptake were found in two patients with an early objective response compared to one patient with disease progression (17). However, this initial study provided only preliminary findings, which need to be confirmed in larger validation studies. In another clinical trial, researchers found that a higher ^18^F-RGD uptake in pretreated tumors predicted better responses to apatinib alone (9). The results of this study were, however, not entirely consistent with previous research. For example, higher RGD uptake values at baseline have been shown to predict poorer outcomes after concurrent chemoradiotherapy in both NSCLC and glioblastoma (18, 19). The primary role of antiangiogenic therapy alone, for example, apatinib, endostar or bevacizumab, may be to prevent new vessels from forming and maintaining tumor vessels in an inactive state, although with different mechanisms of action (20). Integrin αvβ3, which is highly expressed on newly formed vessels, should be reduced by these drugs. It seems therefore that higher RGD uptake can represent a high density of effective target receptors, which increases the sensitivity to antiangiogenic treatments alone and therefore also increases the predictive power.

However, dilated, tortuous, and hyperpermeable angiogenesis represented by RGD uptake values increased tumor interstitial fluid pressure and led to poor blood flow and severe hypoxia, which increased tumor resistance to chemoradiotherapy (19, 21). In this trial, the predominant role of the antiangiogenic drug was to prune tumor vessels and improve the function of the remaining vessels to induce chemopotentiation by enhanced delivery of chemotherapeutic agents according to the vascular normalization theory. In this instance, high RGD uptake not only predicted improved outcomes with antiangiogenic agents but also suggests that the penetrative capacity of chemotherapy is enhanced and therefore can kill tumors with a higher sensitivity.

Previous studies have provided evidence that serum inflammatory factors are associated with immunological status within tumor microenvironments (10). In a retrospective study of patients receiving bevacizumab combined with chemotherapy, researchers found that NLR and PLR in sera decreased in patients with CR/PR but increased in patients with PD, while LMR was elevated in patients with CR/PR and reduced in those with evidence of PD (22). Additionally, low levels of baseline LMR, an increased NLR and a decreased LMR were all independent risk factors for death. Our findings further validate the predictive value of inflammatory factors, confirming that LMR pretreatment is an independent prognostic factor for PFS. This phenomenon may be explained simply because more lymphocytes (and fewer monocytes) migrate into the tumor microenvironment and therefore improve immunological responses, which help to prevent tumor growth. Lymphocytes are thought to play a critical role in immunology by inhibiting tumor cell proliferation and migration (23). Additionally, neutrophils and macrophages, derived from monocytic precursors within tumors, promote angiogenesis. By releasing proangiogenic growth factors such as VEGF and triggering VEGF-independent angiogenesis, this process ultimately leads to antiangiogenic agent resistance (24, 25). Therefore, inflammatory factors certainly appear to be useful predictors of combined antiangiogenic responses.

Although there was no significant correlation between the SUVpeak and PreLMR, the combination of the two parameters may provide a more accurate prognostic value and enable the identification of a subgroup of patients with a high SUVpeak with a low PreLMR and patients with a low SUVpeak with a high PreLMR who encountered intermediate survival. While this study may advance shared decision-making processes allowing patients to plan their futures, it also suggests that intercalating parameters may further increase predictive power.

To the best of our knowledge, this is the first clinical study to apply ^18^F-RGD uptake on PET/CT and the combined biomarker with serum inflammatory factors for selecting optimal advanced NSCLC patients to receive combined antiangiogenic treatment. As such, we ought to reflect on the limitations of this study design and our testing. First and foremost, the sample size was relatively small, and the credibility of the results need to be further verified. Therefore, larger studies are needed, not only to verify the results but also to further develop and validate biomarkers. Additionally, predictors of ^18^F-RGD PET/CT for short-term efficacy and survival were inconsistent. The earlier the effective treatment is determined, the greater the benefit for patients (26); however, the objective response rate did not appear to have a predictive function for PFS in this study. This may have led to inconsistent predictions, and studies with a larger sample size are needed. Second, inflammatory factors may be influenced by other causes, such as comorbid infections and the use of steroids. Even though patients with autoimmune diseases and active infections were excluded from this study, some of our participants may have been in the early stages of developing other conditions.

In conclusion, pretreatment ^18^F-RGD uptake on PET/CT imaging and systemic inflammatory biomarker may predict outcomes of combined antiangiogenic treatment and chemotherapy, and a higher ^18^F-RGD uptake or higher PreLMR may better predict short-term responses and PFS. Combined biomarkers based on molecular imaging with ^18^F-RGD PET/CT and serum inflammatory biomarkers may increase the prognostic value and provide a basis for risk stratification.

## Data Availability Statement

The raw data supporting the conclusions of this article will be made available by the authors, without undue reservation.

## Ethics Statement

The studies involving human participants were reviewed and approved by Shandong cancer hospital. Written informed consent to participate in this study was provided by the participants’ legal guardian/next of kin. Written informed consent was obtained from the individual(s), and minor(s)’ legal guardian/next of kin, for the publication of any potentially identifiable images or data included in this article.

## Author Contributions

JL, LW, and ZL collected the data. JL wrote the manuscript. JNL and ZG helped to collect literature and participated in discussions. JY and XM designed and verified the study. SS helped to write and then edited this report for publication. All authors contributed to the article and approved the submitted version.

## Funding

This study was supported by National Natural Science Foundation of China (81972864); Science and Technology Support Plan for Youth Innovation Teams of Universities in Shandong Province (2019KJL001); Science and Technology Plan of Jinan (201907113); The National Key Research and Development Projects of China (2018YFC1312201); Radiation Oncology Innovate Unit, Chinese Academy of Medical Sciences (2019RU071); the Academic Promotion Program of Shandong First Medical University (2019ZL002) and the foundation of National Natural Science Foundation of China (81972863, 8162790 and 82030082).

## Conflict of Interest

The authors declare that the research was conducted in the absence of any commercial or financial relationships that could be construed as a potential conflict of interest.
