# Supplementary material for: 18F-RGD PET/CT and Systemic Inflammatory Biomarkers Predict Outcomes of Patients With Advanced NSCLC Receiving Combined Antiangiogenic Treatment
Source: Front Oncol. 2021 Jun 4;11:671912. doi: 10.3389/fonc.2021.671912 (PMC8212050; doi:10.3389/fonc.2021.671912)
Supplement: Supplementary file 1 [file Table_1.docx]

**Table S1.** Patients pre-treatment cell counts in the blood and the calculated inflammatory biomarkers

| Patient no. | Pretreatment cell counts in blood (10^9/L) | | | |  | Inflammatory biomarkers | | |
| --- | --- | --- | --- | --- | --- | --- | --- | --- |
|  | Neutrophils | Lymphocytes | Monocytes | Platelets |  | NLR | PLR | LMR |
| 1 | 3.10 | 1.79 | 0.48 | 291.00 |  | 1.73 | 162.57 | 3.73 |
| 2 | 4.90 | 2.01 | 0.36 | 412.00 |  | 2.44 | 204.98 | 5.58 |
| 3 | 11.29 | 0.55 | 1.44 | 427.00 |  | 20.53 | 776.36 | 0.38 |
| 4 | 2.02 | 1.05 | 0.49 | 176.00 |  | 1.92 | 167.62 | 2.14 |
| 5 | 4.07 | 1.52 | 0.58 | 194.00 |  | 2.68 | 127.63 | 2.62 |
| 6 | 4.03 | 1.72 | 0.46 | 222.00 |  | 2.34 | 129.07 | 3.74 |
| 7 | 4.65 | 1.94 | 0.29 | 233.00 |  | 2.40 | 120.10 | 6.69 |
| 8 | 4.53 | 1.63 | 0.38 | 349.00 |  | 2.78 | 214.11 | 4.29 |
| 9 | 4.20 | 1.39 | 1.03 | 257.00 |  | 3.02 | 184.89 | 1.35 |
| 10 | 5.25 | 0.96 | 0.55 | 324.00 |  | 5.47 | 337.50 | 1.75 |
| 11 | 2.45 | 0.85 | 0.32 | 139.00 |  | 2.88 | 163.53 | 2.66 |
| 12 | 5.34 | 1.06 | 0.83 | 167.00 |  | 5.04 | 157.55 | 1.28 |
| 13 | 4.56 | 1.18 | 0.67 | 301.00 |  | 3.86 | 255.08 | 1.76 |
| 14 | 3.15 | 1.17 | 0.26 | 257.00 |  | 2.69 | 219.66 | 4.50 |
| 15 | 4.76 | 1.30 | 0.62 | 350.00 |  | 3.66 | 269.23 | 2.10 |
| 16 | 4.20 | 0.60 | 0.41 | 260.00 |  | 7.00 | 433.33 | 1.46 |
| 17 | 4.91 | 2.84 | 0.92 | 190.00 |  | 1.73 | 66.90 | 3.09 |
| 18 | 6.25 | 1.94 | 0.65 | 285.00 |  | 3.22 | 146.91 | 2.98 |
| 19 | 3.25 | 0.78 | 0.35 | 309.00 |  | 4.17 | 396.15 | 2.23 |
| 20 | 12.48 | 2.35 | 1.02 | 296.00 |  | 5.31 | 125.96 | 2.30 |
| 21 | 8.73 | 0.70 | 0.14 | 297.00 |  | 12.47 | 424.29 | 5.00 |
| 22 | 4.13 | 0.56 | 0.84 | 147.00 |  | 7.38 | 262.50 | 0.67 |
| 23 | 6.19 | 2.14 | 0.87 | 609.00 |  | 2.89 | 284.58 | 2.46 |
